# Uric acid, as a double-edged sword, affects the activity of epidermal growth factor (EGF) on human umbilical vein endothelial cells by regulating aging process

**DOI:** 10.1080/21655979.2022.2027172

**Published:** 2022-02-13

**Authors:** Yuan Li, Linru Zhao, Wufang Qi

**Affiliations:** Department of Rheumatology and Immunology, Tianjin First Central Hospital, Tianjin, People’s Republic of China

**Keywords:** Uric acid, EGF, HUVEC, aging, signaling transduction

## Abstract

Uric acid (UA) is the main metabolite of the human body. Although UA is only a product of metabolism, it is important biological regulator. Epidermal growth factor (EGF) has important biological functions. However, so far, the effect of UA on EGF’s activity has not been revealed. For this, in the current study, we systematically studied the effect of OA on the biological activity of EGF. Human Umbilical Vein Endothelial Cells (HUVECs) were used as an *in vitro* model, and Western-blot, RT-PCR, laser scanning confocal microscopy (CLSM) and co-localization analyses were carried out. The results showed that high concentration of UA (10 mg/dl) severely affected the biological activity of EGF. High concentration of UA suppressed the activity of EGF, and inhibited the biological effect of EGF on the HUVECs. However, it is interesting that EGF-mediated intracellular signaling was significantly down-regulated in the H_2_O_2_-induced senescent HUVEC, and physiological concentration of UA could at least partially restore the EGF-mediated signaling. Further work showed that physiological concentration of UA (5 mg/dl) shows the anti-aging effect. Taken together, current research indicates that UA may be a ‘double-edged sword’, physiological concentration of UA may be beneficial, but high concentrations of uric acid (UA) are harmful.

## Introduction

Uric acid (UA) is the end-product of purine nucleotide catabolism, and the level of UA exceeding the physiological level becomes the potential risk factor for many diseases [1]. Hyperuricemia refers to the serum uric acid concentration (males>416.4 μmol/L, 7.0 mg/dL; females>356.9 μmol/L, 6.0 mg/dL), the diagnostic criteria for hyperuricemia are slightly different in different countries and regions. The excess UA in the body can cause urate crystals that will be deposited in joints and tissues [2]. Hyperuricemia is not only the main cause of gout and kidney stones but also a predisposing factor of cardiovascular diseases (such as hypertension and metabolic syndrome) [3]. The endothelial function of blood vessels has an important protective effect on the cardiovascular system [4]. However, high UA can lead to damage to the vascular endothelial system [5]. UA is an antioxidant. But under certain conditions, UA can be converted from antioxidants to pro-oxidants, which could result in oxidative stress and damage to the vascular endothelium [6]. In addition, UA can also induce inflammation [6]. A series of studies have reported that high UA leads to endothelial damage, which in turn causes endothelial dysfunction. Previous study reported that high UA can cause oxidative stress and inflammation [7], and oxidative stress and inflammation are important potential risk factors for vascular endothelial cell damage. Therefore, how to reduce the vascular endothelial damage caused by hyperuricemia and protect the vascular function is an important scientific problem.

The growth development of vascular endothelial cell is closely related to the endocrine system, which are regulated by a variety of cytokines and growth factors. Among them, EGF is one of the most important cytokine. EGF is a multifunctional cytokine, and its function is exhibited by binding to its receptor (Epidermal growth factor, EGFR) [8]. After EGF binds to EGFR, EGFR-mediated downstream signaling pathways are triggered, these signaling pathways work together to contribute to a series of physiological effects, such as regulating cell proliferation. It has been reported that EGF has the important biological effects on vascular endothelial cells [9]. Studies have found that EGF can promote the proliferation of vascular endothelial cells [10]. Furthermore, EGF could regulate monolayer permeability of HUVECs [11]. In addition, EGF could promote the HUVEC tube formation [12].

In the current research, we have studied the following three important scientific issues: 1) we have investigated EGF/EGFR’s cell behavior on HUVEC, and found that EGF/EGFR could transport into the cell nuclei. Further study showed that nuclear EGFR could regulate HUVEC proliferation; 2) we studied the effect of high concentration of UA on the activity of EGF/EGFR, and found that the high concentration of UA inhibited the activity of EGF/EGFR in HUVECs. However, the low concentration of UA had no obvious effect on the activity of EGF/EGFR; 3) we explored the potential mechanism by which high concentration of UA effects the activity of EGF/EGFR system, and found that high concentration of UA induced the senescence of HUVEC, which may be underlying mechanism leading to EGF/EGFR system dysfunction. Additionally, we found that physiological concentration of UA could not induce HUVEC senescence. On the contrary, physiological concentration of UA could inhibit the HUVEC senescence, but high concentration of UA could promote HUVEC senescence. Taken together, the current research lays the foundation for further research on the regulatory effect of EGF-EGFR system on HUVEC.

## Materials and methods

### Materials

Uric acid (UA) was obtained from Sigma–Aldrich (Carlsbad, CA). Uric acid powder was dissolved in a 1 mol/L NaOH solution at a concentration of 40 mmol/L. P16 (catalog no. ab51243, 1:200 dilution), p21 (catalog no. ab109520, 1:100 dilution) antibodies were purchased from Abcam company (UK). BCA Protein Concentration Kit (catalog no. PC0020), Hoechst 332,342 staining kit (catalog no. C0021), DAPI and trypsin were purchased form Solarbio company (China). RIPA lysate, glutathione peroxidase (GSH-PX) assay kit, reactive oxygen assay species assay kit were purchased from Beyotime Biotechnology company (Shanghai, China). BSA (Bovine Serum Albumin), DMEM culture medium and Fetal bovine serum (FBS) were purchased from Thermo Fisher (USA). An enhanced chemiluminescence (ECL) kit and Polyvinylidenefluoride (PVDF) membrane were obtained from Merck Millipore (Massachusetts, USA). 4% paraformaldehyde (catalog no. P6148), Triton X-100 (catalog no. 93,443) were purchased from Sigma-Aldrich (USA). Clathrin monoclonal antibody (catalog no. ab21679, 1:500 dilution) and Caveolin monoclonal antibody (catalog no. ab87770, 1:1000 dilution) were purchased from Abcam (USA). p-JAK2monoclonal antibody (catalog no. 3771, 1:500 dilution), p-STAT5 monoclonal antibody (catalog no. 4322, 1:500 dilution), β-actin (catalog no. 3700, 1:200 dilution), HRP-conjugated anti-rabbit IgG (catalog no. 7074, 1:3000 dilution) were purchased from CST (USA). Unless otherwise specified, other reagents were purchased from Sigma-Aldrich.

### Cell culture

Human umbilical vein endothelial cell (HUVEC) was obtained from Thermo-Fisher (*#*C01510C). Cell were cultured in DMEM (Dulbecco’s Modified Eagle Medium) medium supplemented with 10% fetal bovine serum (FBS), 100 mg/mL streptomycin and 100 U/mL penicillin at 37°C with 5% CO_2_.

### Indirect immunofluorescence assay (IFA)

The experiments were divided into the following groups: 1) control group (n = 5); 2) High concentration of UA group (n = 5); 3) physiological concentration of UA group (n = 5). When the HUVEC density reached 50% confluence, the cells were serum-starved for 6 h. After washing for three times with PBS, the cells were fixed with 4% paraformaldehyde for 30 min at RT. The cells were treated with 0.1% Triton X-100 for 5 min to permeate the cell membrane at RT. After three washes with PBS, the cell samples were blocked with 5% BSA for 2 h at RT. The cells were washed 3 times in PBS, and incubated with the primary antibodies as indicated in the figure legends at 4°C overnight, followed by treatment with the appropriate fluorescence-labeled secondary antibody at 37°C for 60 min in the dark. After washing, the cells were incubated with Hoechst 33,258 to stain the cell nuclei in the dark at 37°C for 10 min. The cells were observed with a confocal laser scanning microscope (CLSM, Leica-STELLARIS). Image J software was used for image analysis.

### MTT assays

HUVECs were seeded into a 96-well plate at a density of 1 × 10^4^ cells/well. The cells were cultured in serum-free medium for 12 h. Cells were then treated with EGF (30 ng/mL) for the indicated time points, and cell proliferation was measured by the MTT solution (1 mg/mL). The optical density (OD) at 450 nm was measured with a microplate reader (Thermo Scientific, Multiskan FC). Cell growth was analyzed using the cell-IQ system. The cell growth curve was drawn according to Cell-IQ system assays.

### Measurement of cytokines by ELISA

Immunoreactive TNF-α (catalog no. SEKM-0034, Salorbio life science) and IL-6 (catalog no. SEKM-007, Salorbio life science) were measured by ELISA kits according to the manufacturer’s instructions.

### Western-blot analysis

Western blotting analyses were conducted to determine the levels of various proteins.

In brief, cells grew to approximately 80% confluence were treated with EGF, and incubated at 37°C for the indicated time points. After EGF treatment, the cell lysates were centrifuged and the cell supernatant was collected. The protein concentration was determined by the BCA kit, equal amount of protein (30 μg) were resolved via SDS-PAGE and transferred to the PVDF membranes (Millipore). After washing, the PVDF membranes were incubated with various primary antibodies overnight at 4°C, followed by treatment with the appropriate fluorescence-labeled secondary antibody. The immuno-blots were visualized by Bio-rad system (ChemiDoc XRS) (or by the ECL system). Equal loading was determined via β-actin (or GAPDH) levels.

### Reverse transcription quantitative polymerase chain reaction (RT-qPCR)

Trizol was used to isolate total RNA from the HUVECs according to the manufacturer’s instructions. The RNA concentration was determined by ultraviolet spectrophotometer. RNA was reverse transcribed into cDNA, RT-qPCR was then performed using the SYBR®Premix Ex TaqTM Kit on the ABI7500 quantitative PCR instrument (Thermo-Fisher Scientific Inc.). The relative expression level of mRNA was normalized to GAPDH and was calculated using the 2-ΔΔCt method. The primer sequences were shown as follows: GAPDH (human): Forward primer:ACCACAGTCCATGCCATCAC, Reverse TCCACCACCCTGTTGCTGTA; Human-TNF-α 5-CTCGAACCCCGAGTGACAAG; R:5’-TGAGGTACAGGC CCTCTGAT3’; IL-6: F:5’-GCTCCAGTTGCCTTTCTTCCC-3’; R:5ʹGTGCCTCTTTGCTGCTTTC-3’.

### Cell cycle analysis

The cells were treated with EGF as indicated in the figure legends. The cells were then harvested. 70% ethanol were added to fix the cell. After washing, RNase A enzyme was added, after which, 1 ml PI staining solution (50 μg/ml) was added and incubated in the dark at 4°C for 0.5 h. The cell samples were then analyzed by Flow cytometry (BD Accuri C6).

### Cell apoptosis analysis

HUVECs were then digested with 0.25% trypsin, the cells were collected by centrifugation at 1000 r/min for 5 min. After washing, the cells were double-stained with annexin V/PI and tested with flow cytometry (BD Accuri C6).

### Establishment of a senescent HUVEC model

HUVECs were treated with H_2_O_2_ to establish the senescence model. HUVECs were exposed to various concentrations (10–50 μmol/L) of H_2_O_2_ to induce HUVECs senescence. After H_2_O_2_ treatment, the cells were harvested for further assessment by detecting the aging-related marker molecules.

### β-gal staining

The cells were seeded on a 6-well cell culture plate and cultured into 70% confluence. After washing with PBS, 1 ml of fixative (4% PFA) was added to fix the cell at RT for 15 min. After washing the cells twice, the cell fixation solution was discarded, and the cells were washed 3 times with PBS. Then, β-gal staining solution was added and incubated overnight at 37°C, and then cell samples were observed under an optical microscope.

### Statistics analysis

All results were expressed as mean ± standard deviation (SD). Statistical analyses were performed using Prism (8.2.0) software (GraphPad; San Diego, CA). Statistical significance was determined by a Student’s t-test, A P-value less than 0.05 was considered statistically significant.

## Results

In this work, we have investigated the following three important scientific issues: 1) we have systematically investigated EGF/EGFR’s cell characteristics on HUVEC, and found that EGF/EGFR could transport into the cell nuclei. Further experiments showed that nuclear EGFR could promote HUVEC proliferation; 2) we studied the effect of high concentration of UA on the activity of EGF/EGFR, and found that the high concentration of UA inhibited the activity of EGF/EGFR in HUVECs. However, the low concentration of UA had no obvious effect on the activity of EGF/EGFR; 3) we explored the potential mechanism by which high concentration of UA effects the activity of EGF/EGFR system, and found that high concentration of UA induced the senescence of HUVEC, which may be underlying mechanism by which EGF/EGFR-mediated signaling was down-regulated. It is interesting that physiological concentration of UA could not induce HUVEC senescence. However, physiological concentration of UA could inhibit the HUVEC senescence induced by H_2_O_2_, but high concentration of UA could promote HUVEC senescence possible through induction of oxidative stress and inflammation. Taken together, the current research lays the foundation for further research on the regulatory effect of EGF-EGFR system on HUVEC.

### Analysis of EGF/EGFR’s cell behavior in HUVECs

The cell behavior of EGF/EGFR in the HUVEC has not yet been fully revealed. Therefore, we explored the cell characteristics of EGF/EGFR in the HUVEC model. Firstly, we checked the EGFR expression, Figure 1(a) showed that EGFR mainly expressed on the cell membrane and a small amount of EGFR was localized in the cytoplasm. Next, the internalization of EGF was analyzed by CLSM, and the results showed that EGF internalized into HUVEC in a time-dependent manner. The fluorescent signal could be detected in the cell membrane of HUVECs after EGF exposure for 1 min. With the extension of EGF exposure time, the intracellular fluorescent signal was constantly increased (15–60 min). After EGF exposure for 60 min, the fluorescent signal of

**Figure 1.**
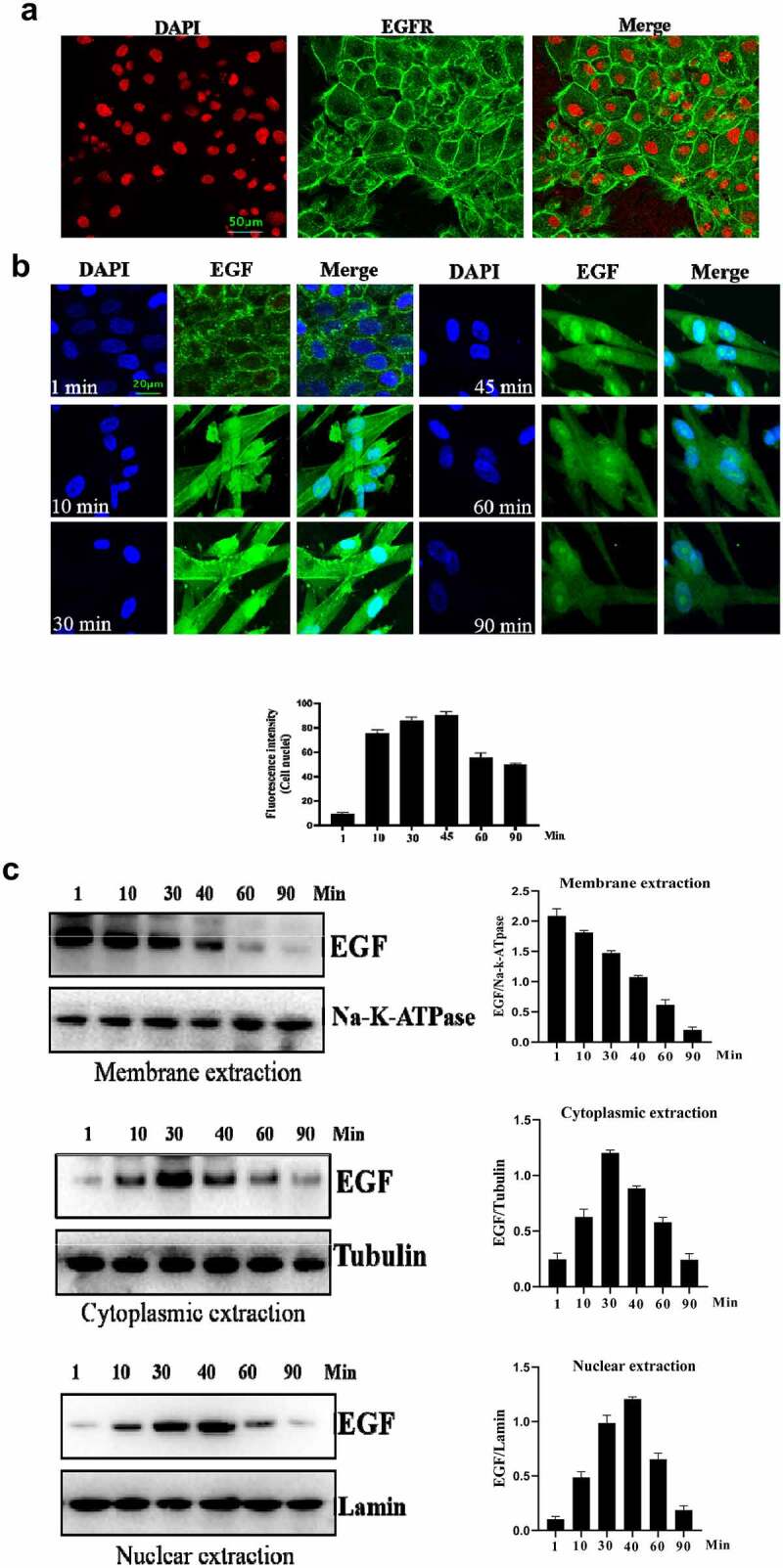
Evaluation of the intracellular trafficking of EGF/EGFR.a. Analysis of EGFR expression on HUVECs. b. HUVECs were seeded onto the glass coverslips and cultured for 12 h. After three washes with PBS, the cells were fixed with 4% PFA for 10 min at 37°C. After washing, the cells were then permeabilized with 90% ice-cold methanol for 15 min at −20°C. The cells were then incubated with 5% BSA to block cell samples. The cells were then treated with the indicated anti-EGFR antibody(Abcam, #ab52894, 1:200 dilution). After three washes, the cells were incubated with secondary antibody (IgG H&L (Alexa Fluor® 488), #ab150077, 1:1000 dilution). After three washes, the cell samples were detected by CLSM (Leica-STELLARIS). b. Internalization of EGF in HUVECs. HUVECs were seeded on the glass coverslips and cultured for 12 h. After the cells were cultured in serum-free medium for another 6 h. The HUVECs were incubated with EGF (30 ng/mL) for various time periods. AfterEGF treatment, the cells were fixed and cell nuclei were stained with DAPI. c. The subcellular localization of EGF in HUVECs was analyzed by Western-blot. d. HUVECs were challenged with EGF (30 ng/mL) for 0–120 min. After EGF exposure, the cells were fixed with 4% PFA and permeabilized with 0.5%triton X-100. After blocking with 5% BSA, the cells were labeled with a rabbit monoclonal antibody against EGFR (1:200 in PBST containing 4% BSA) overnight at 4°C. a fluorescently conjugated goat anti-rabbit IgG was used as the secondary antibody to stain the cells. The cells were observed by CLSM. e. The subcellular localization of EGFR in HUVECs was analyzed by Western-blot. Data are presented as mean ± SD (n = 5).

**Figure 1b.**
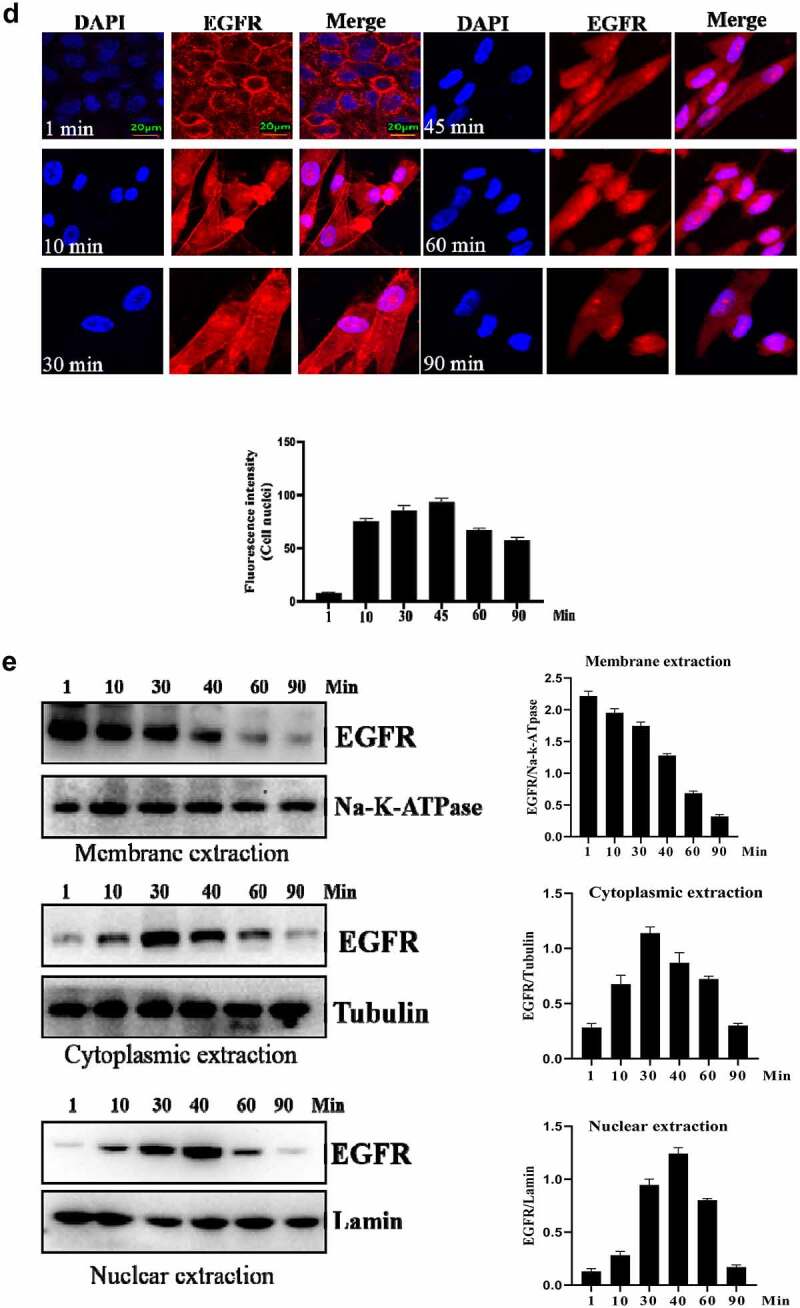
Continue

the cell started to decline (Figure 1(b)). Furthermore, Western blot analysis also confirmed the findings of IFA (Figure 1(c)).

Next, the internalization of EGFR was investigated under EGF stimulation. As shown in Figure 1(d), EGF stimulation could induce the internalization of EGFR. Without stimulation, EGFR was mainly located in the cell membrane. After EGF treatment, EGFR began to internalize into cell, the internalization of EGFR induced by EGF showed a time-dependent manner. An interesting phenomenon is that EGFR transported into the cell nuclei. Furthermore, Western-blot analysis also confirmed the findings of IFA (Figure 1(e)).

### Nuclear-localized EGFR is involved in HUVEC proliferation

Firstly, we analyzed the EGFR’s nuclear transport pathway, it can be seen that EGFR’s endocytosis was mediated by clathrin and/or caveolin by IFA and IP-WB assays (Figure 2(a)). Further experiments showed that the internalized EGFR entered into EEA1-positive endosome (please see supplementary Figure S1). We tried to study the functions of nuclear EGFR. It has been reported that NUP-358 is involved in EGFR’s nuclear localization. Therefore, we established the cell model that EGFR could not transport into cell nuclei under EGF stimulation. It can be seen that the nuclear localization of EGFR was significantly down-regulated after NUP358 knockdown (Figure 2(b)). The effect of NUP358 knockdown on HUVEC was analyzed, and the results showed that the EGFR expression pattern and EGFR expression level were not significantly affected (Figure 2(c)). In addition, NUP-358 knockdown did not affect the internalization and cytoplasmic transport of EGF/EGFR, but NUP-358 knockdown inhibited the EGFR’s nuclear transport (Figure 2(d)). Additionally, knockdown of NUP-358 has no effect on HUVEC viability by MTT and CCK8 analyses (Figure 2(e) and supplementary Figure S2).

**Figure 2.**
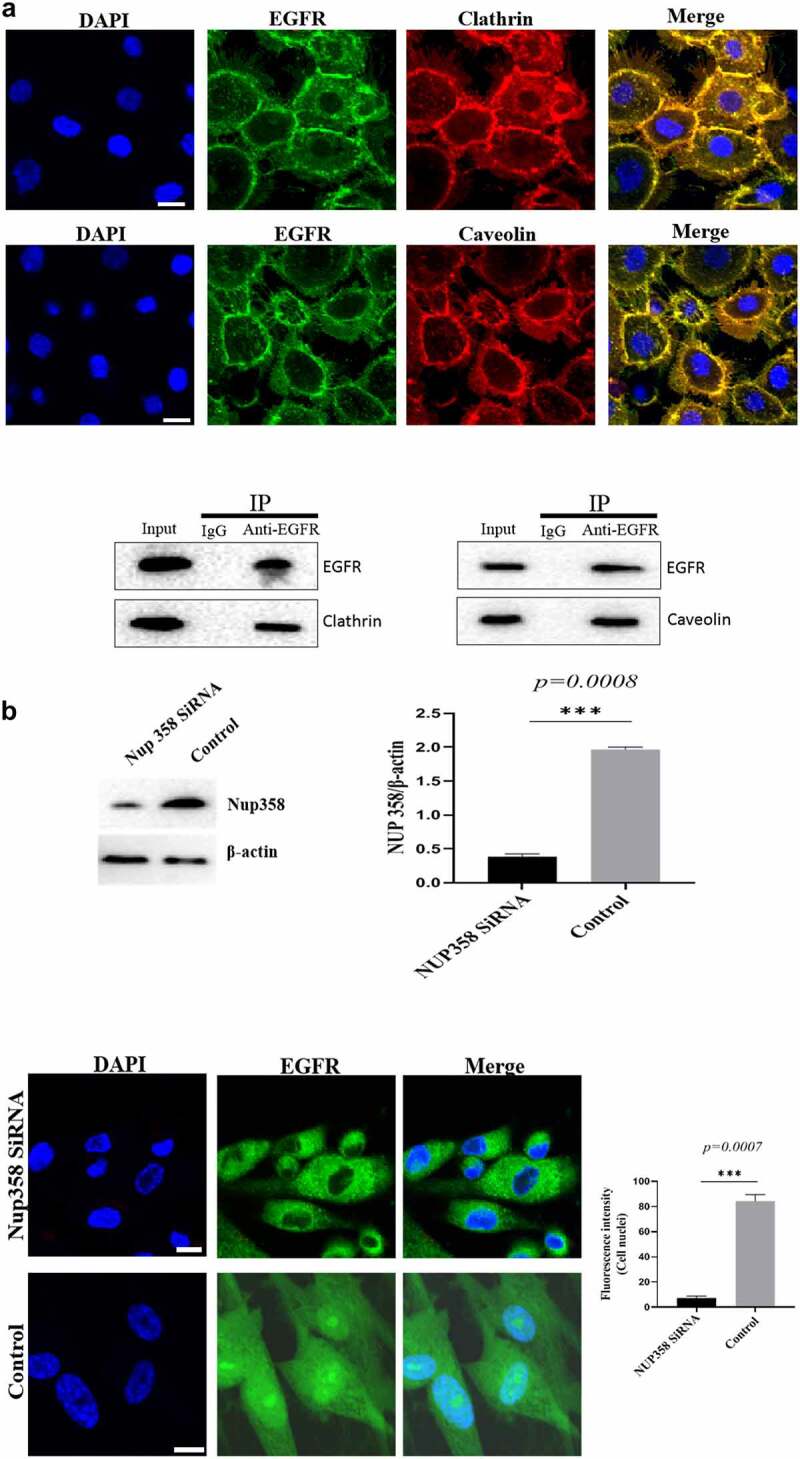
Analysis of the endocytosis of EGF/EGFR. a. EGFR’s endocytosis was mediated via clathrin and/or caveolin by co-localization and IP-WB analysis. b. NUP358 knock-down inhibited the EGFR’s nuclear localization. c.NUP358 knock-down did not affect the EGFR expression pattern and EGFR expression level. d. NUP-358 knockdown did not affect the internalization and cytoplasmic transport of EGF/EGFR. e. Knockdown of NUP-358 had no effect on HUVEC viability by MTT analysis. Data are presented as mean ± SD (n = 5). Asterisks indicate statistically significant differences.

**Figure 2b.**
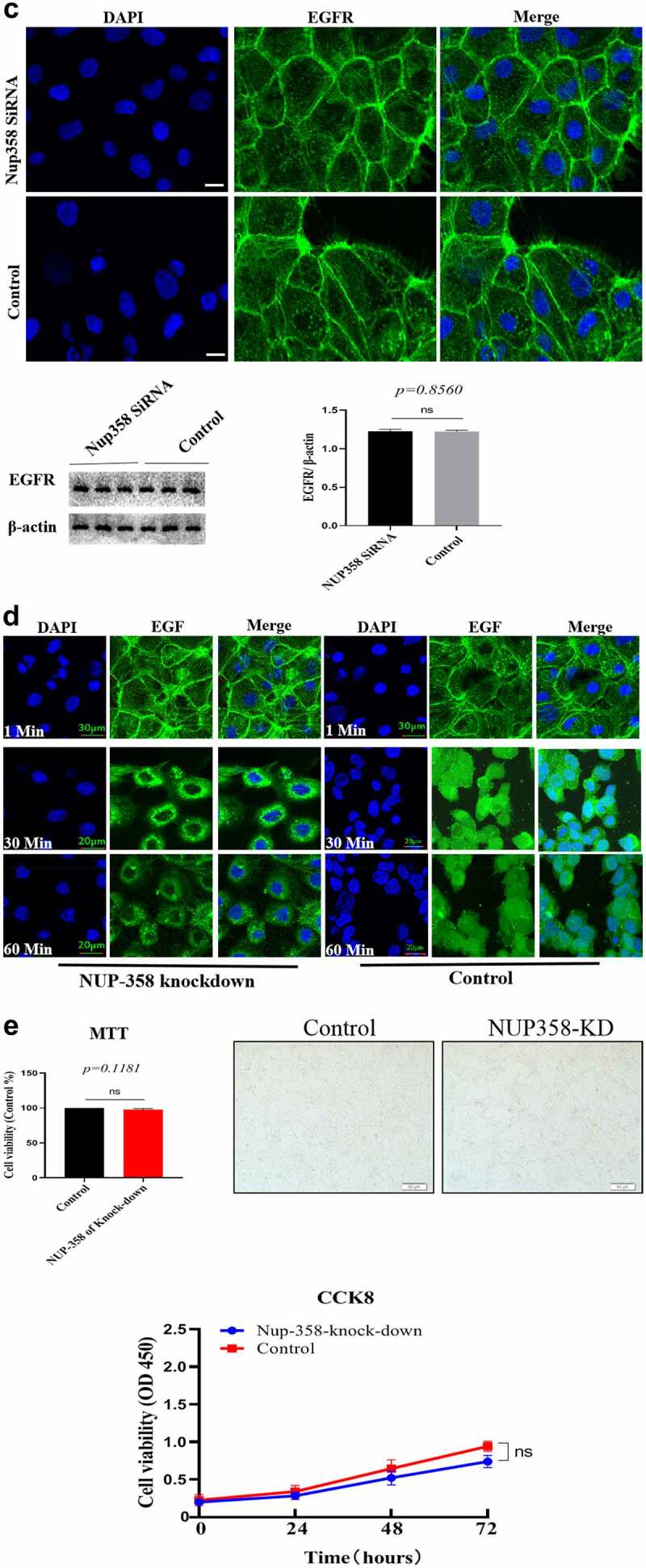
Continue

After successfully establishing the non-nuclear-localized EGFR’s cell model, we then studied the potential functions of nuclear-localized EGFR, as shown in Figure 3(a) and supplementary Figure S3, we found that HUVECs proliferation was significantly reduced compared to EGFR-nuclear-targeted group. Further experiments showed that nuclear EGFR promoted the cell cycle (Figure 3(b)), and the expression of cyclinD1, CDK4, Ki67 and Rb was down-regulated compared to EGFR-nuclear-targeted group (Figure 3(c)).

**Figure 3.**
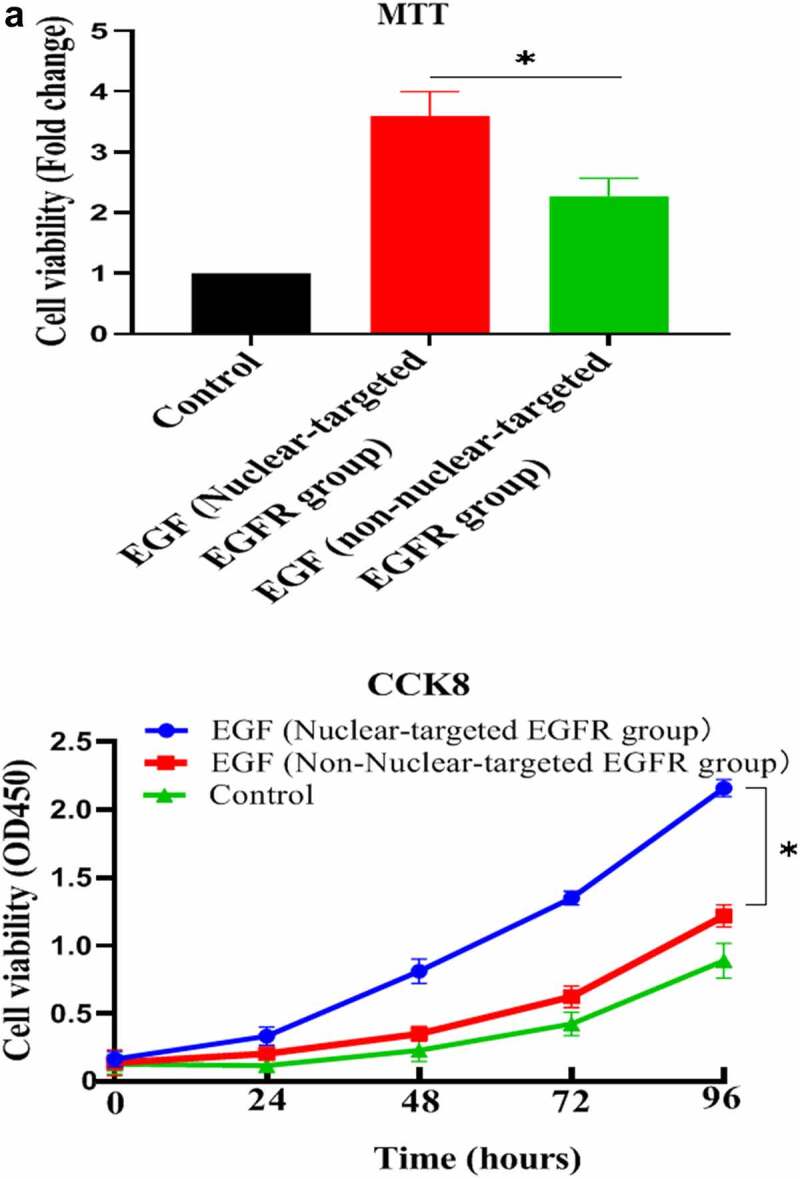
Evaluation of the biological activity of nuclear EGFR. a. HUVEC proliferation were significantly reduced in non-EGFR nuclear localization group by MTT analysis. b. The nuclear-localized EGFR is involved in the cell cycle. c. the expression of cyclinD1, CDK4, Ki67 and Rb were down-regulated.Data are presented as mean ± SD (n = 5). Asterisks indicate statistically significant differences.

**Figure 3b.**
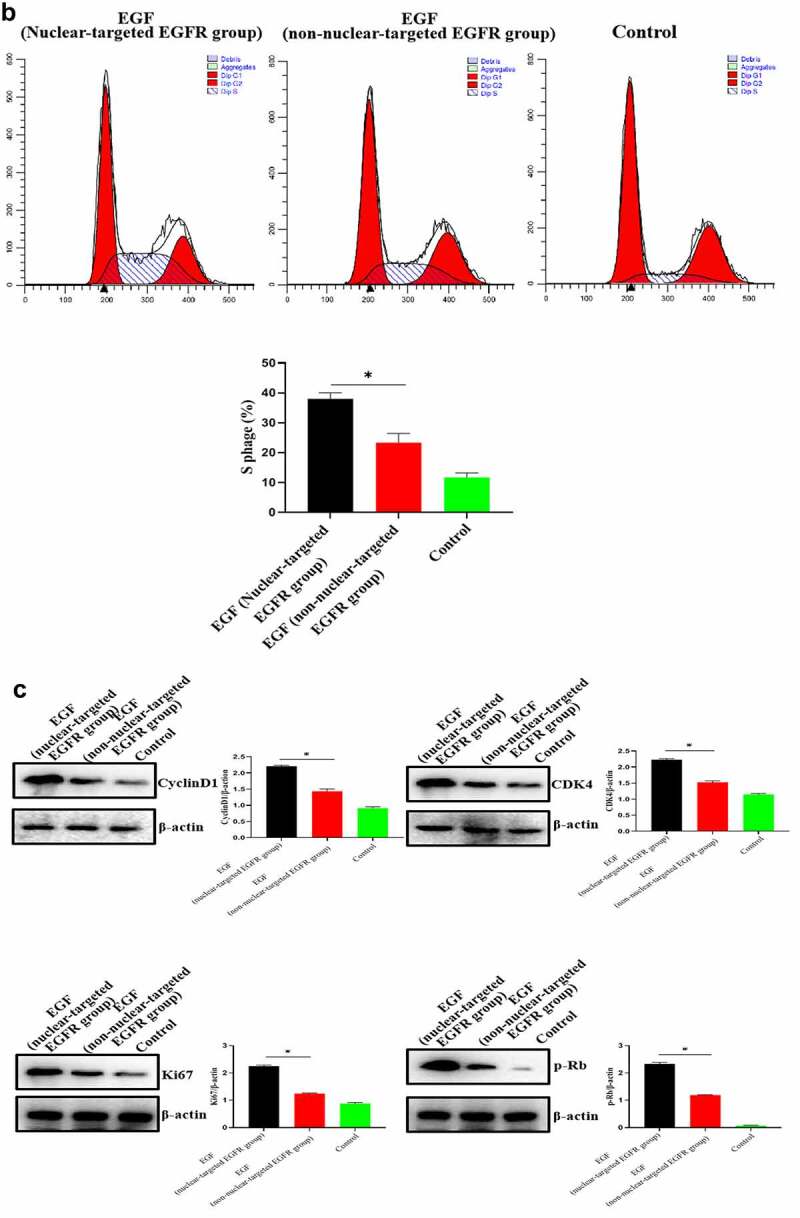
Continue

### The effect of Uric acid on the biological activity of EGF/EGFR in HUVEC

The EGF/EGFR system has an important regulatory effect on the biological activity of HUVEC (such as cell proliferation). Here, we studied the effect of different concentrations of UA on the biological activity of EGF/EGFR. We used two concentrations of UA: 1) the physiological concentration of UA, 5 mg/dL (300 µmol/L); 2) high concentration of UA, 10 mg/dL (600 µmol/L). MTT and CCK8 experiments showed thatUA (5 mg/dl) had no significant effect on the EGF-induced HUVEC proliferation. However, high concentrations of UA inhibited EGF-induced HUVEC proliferation (Figure 4(a) and supplementary Figure S4). Further experiment demonstrated that the expression levels of cyclinD1, p-Rb, Ki67 and CDK4 were also significantly down-regulated compared with the physiological concentration of UA group (5 mg/dl) (Figure 4(b)). On this basis, we further carried out experiments to study the effect of UA on EGF/EGFR signaling, and the results showed that 5 mg/dl did not affect EGF-mediated intracellular signaling, but high concentration of UA (10 mg/dl) severely disrupted the signaling profile of EGF/EGFR (Figure 4(c)). In addition, we also found that UA (10 mg/dl)severely affected the sub-cellular localization of EGFR, which inhibited the nuclear localization of EGFR (Figure 4(d)). These results indicate that high concentrations of UA not only inhibited the EGFR-mediated signaling pathway but also affected the cell behavior of EGFR.

**Figure 4.**
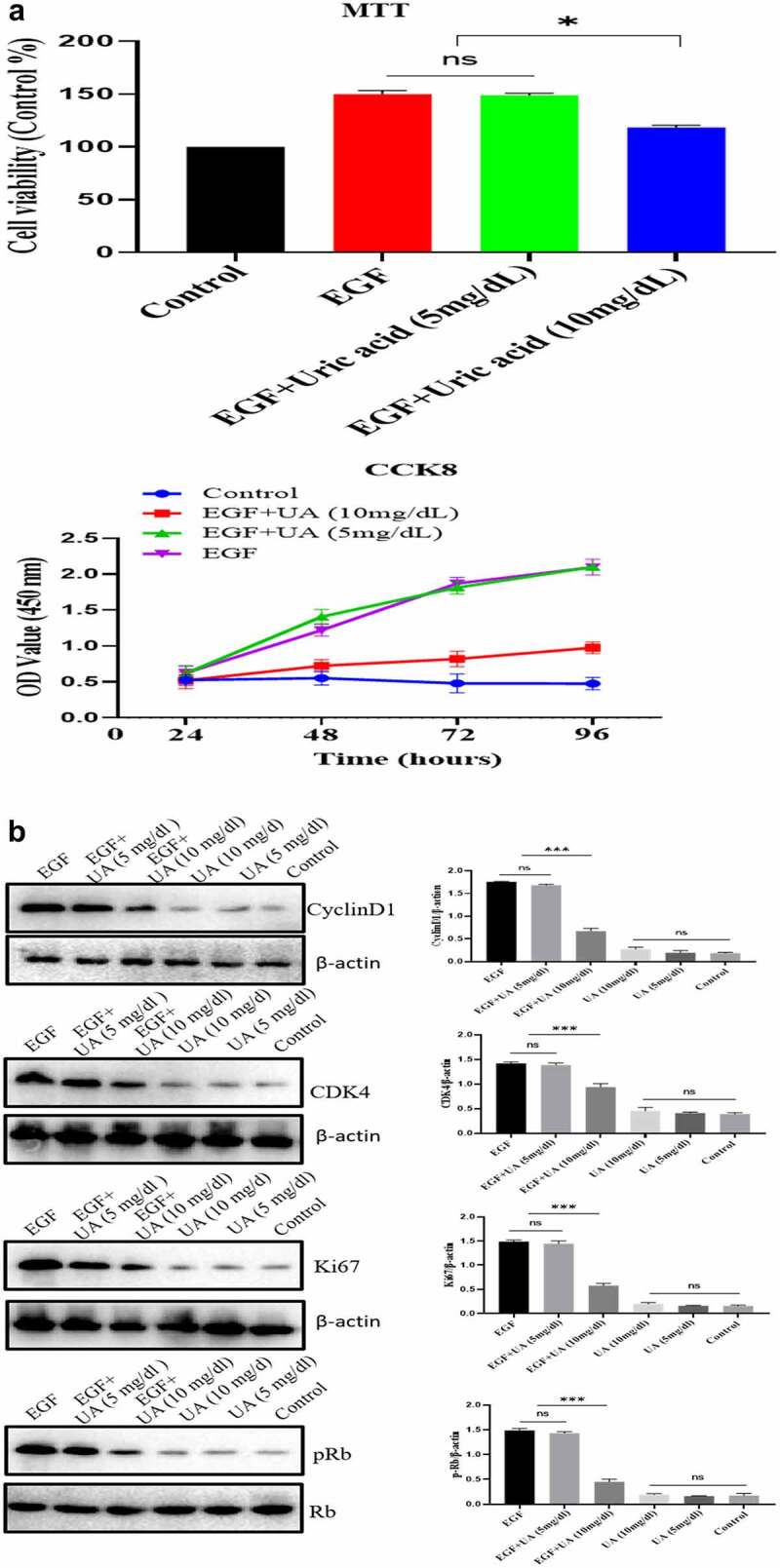
Evaluation of the biological activity of nuclear EGFR. a. Physiological concentration of UA (5 mg/dl) has no effect on the EGF-induced HUVEC proliferation. The cells were pre-treated with high UA (10 mg/dl) or physiological concentration of UA (5 mg/dl) for 24 h, after which, the cell proliferation was analyzed by MTT according to the manufacturer’s instructions. b. EGF-induced expression of cyclinD1, p-Rb, Ki67 and CDK4 was also significantly reduced by high concentration of uric acid(10 mg/dl). c. high concentration of UA (10 mg/dl) severely disrupted the signaling of EGF/EGFR in HUVEC cells model. d. UA (10 mg/dl) inhibited the nuclear localization of EGFR. Data are presented as mean ± SD (n = 5).Asterisks indicate statistically significant differences.

**Figure 4b.**
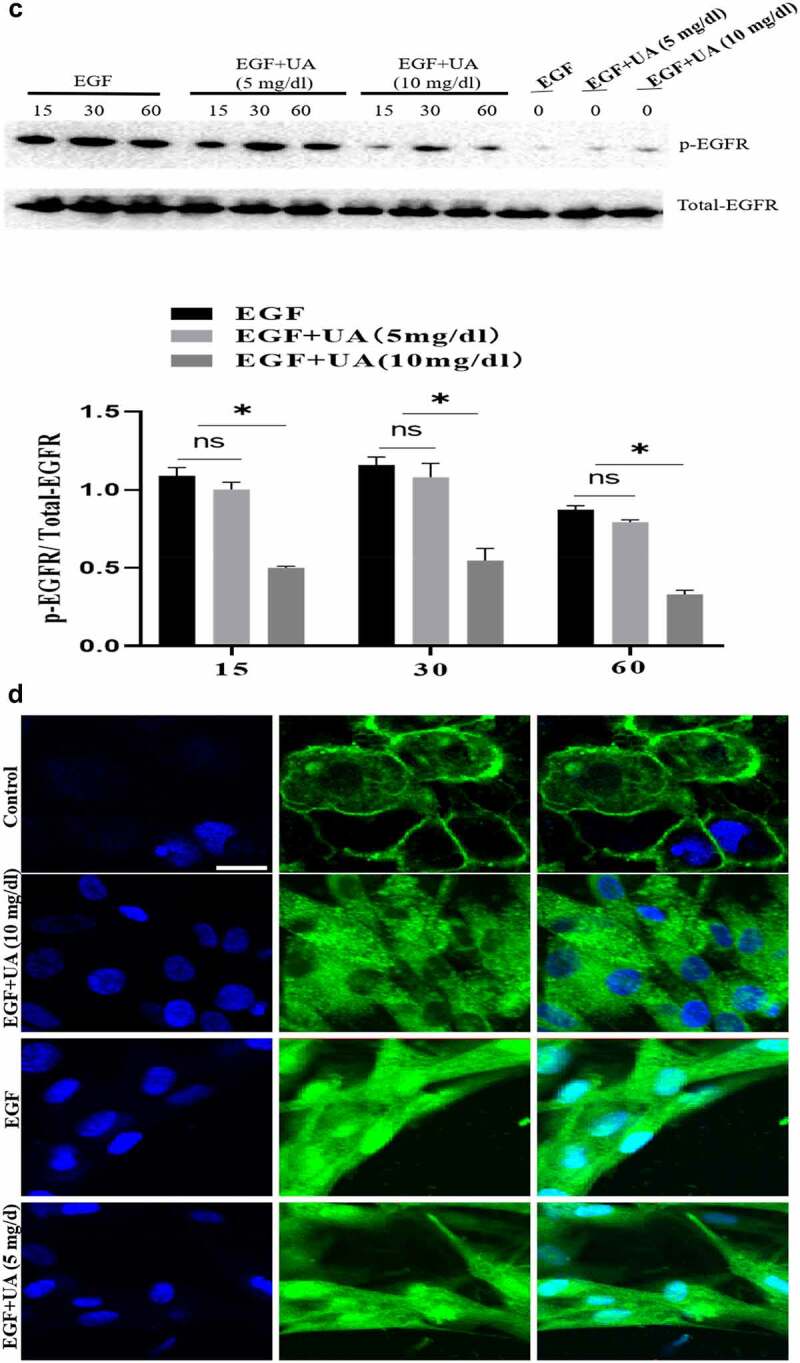
Continue

### High concentration of UA induces the senescence of HUVECs

The above studies showed that high UA (10 mg/dl) inhibited the biological activity of EGF/EGFR. Here, we studied the reason for why high UA inhibits the activity of EGF/EGFR from the perspective of cell senescence, because previous studies have showed that high UA leads to inflammation and oxidative stress, which are potential risk factors causing cell senescence. Based on this, we further analyzed whether high concentration of UA (10 mg/dl) could induce HUVEC senescence, and the results showed that high concentration of UA (10 mg/dl) induces HUVEC senescence compared with the control group by measuring the following aging-related markers: SA-β-gal, p15 and p21. In addition, physiological concentration of UA did not cause HUVEC senescence (Figure 5(a)). Ki67 expression was down-regulated (Figure 5(b)). High concentration of UA (10 mg/dl) up-regulated the expression of inflammatory genes (Figure 5(c)). In addition, high UA (10 mg/dl) caused the oxidative stress of HUVEC (Figure 5(d)). High UA caused cell apoptosis (Figure 5(e)), and cell cycle was changed by UAtreatment (Figure 5(f)). Previous studies have demonstrated that aging could significantly reduce EGF/EGFR signaling [13].

**Figure 5.**
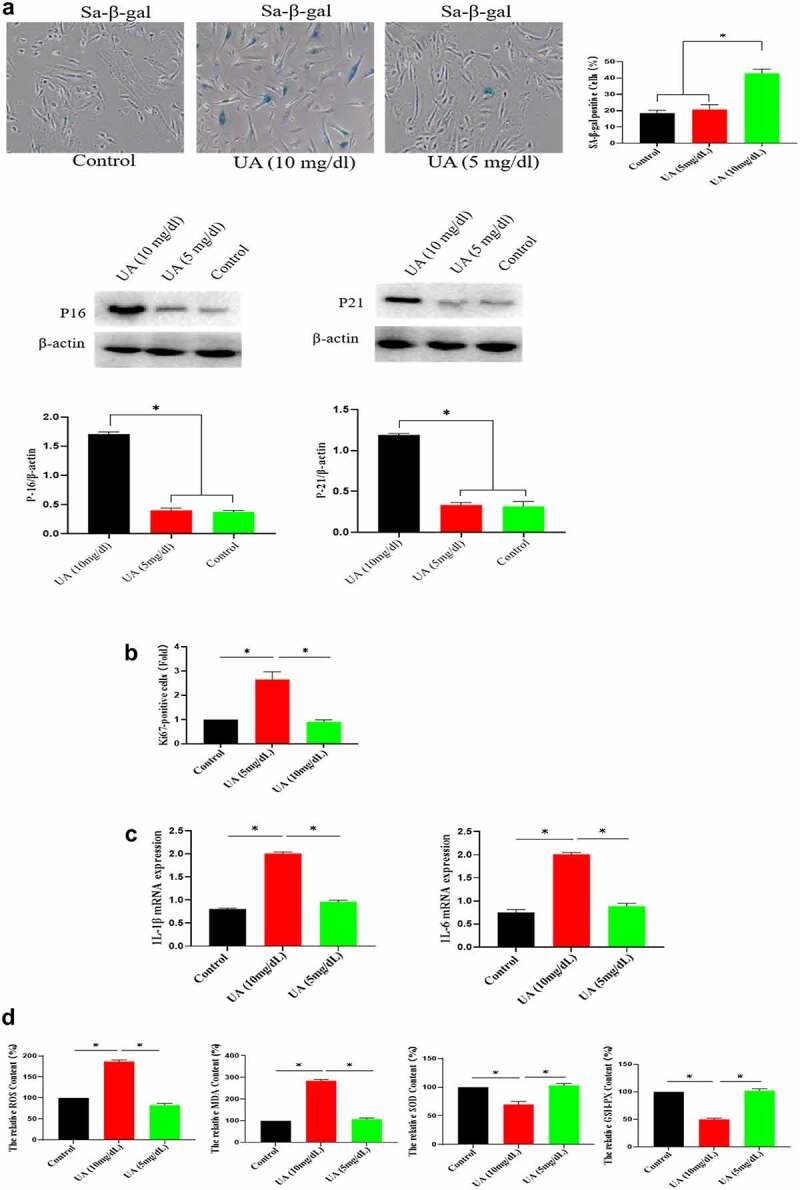
Effect of UA on the HUVEC senescence. a. High concentration of UA induced HUVEC senescence by detecting aging-related markers. b. Ki67 expression was down-regulated by high concentration of UA (10 mg/dl) treatment. c. High concentration of UA (10 mg/dl) increased the expression of inflammatory genes. d. High UA (10 mg/dl) caused the oxidative stress of HUVEC. E-F. High UA caused cell apoptosis and cell cycle changes. The experimental process has been described in detail in Materials and Methods section. Data are presented as mean ± SD. Asterisks indicate statistically significant differences.

**Figure 5b.**
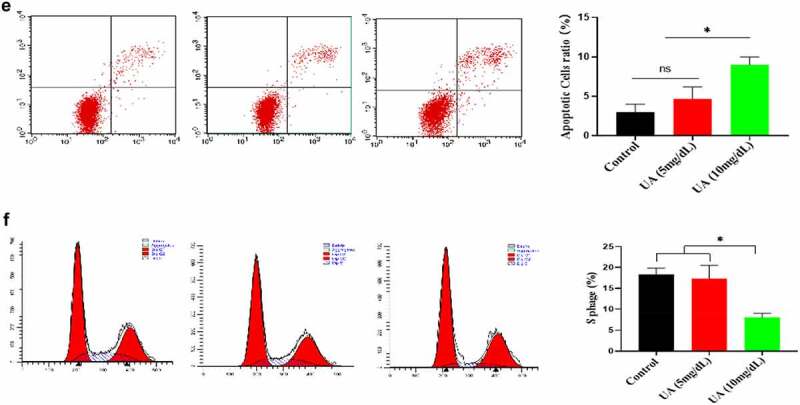
Continue

### Physiological level of UA exhibits anti-aging effect

The above studies have shown that high UA could induce HUVEC senescence, but a large number of literatures have reported that UA itself is an antioxidant molecule, and oxidative stress is an important inducing factor of aging. Therefore, we further analyzed the effect of physiological concentration of UA on H_2_O_2_-induced HUVEC senescence. We first constructed a cell senescence model by H_2_O_2_ treatment, and then uric acid (5 mg/dl) was added and incubated for 24 h, the results showed that physiological concentration of UA (5 mg/dl) has anti-aging effects in the senescent HUVEC (Figure 6(a)). It can be seen that physiological concentration of UA (5 mg/dl) down-regulated H_2_O_2_-induced oxidative stress (Figure 6(b)) and inflammation (Figure 6(c)). Pre-treatment of physiological concentration of uric acid (5 mg/dl) could also restore the signaling ability of EGF/EGFR in the senescent HUVEC (Figure 6(d)). The current study showed that uric acid (UA) is actually a ‘double-edged sword’. At physiological concentration, UA (5 mg/dl) shows anti-aging effect and regulates the biological activity of EGF/EGFR. In contrast, high concentration of UA is a potential risk factor, which not only induces cell senescence but also leads to signaling down-regulation of EGF/EGFR.

**Figure 6.**
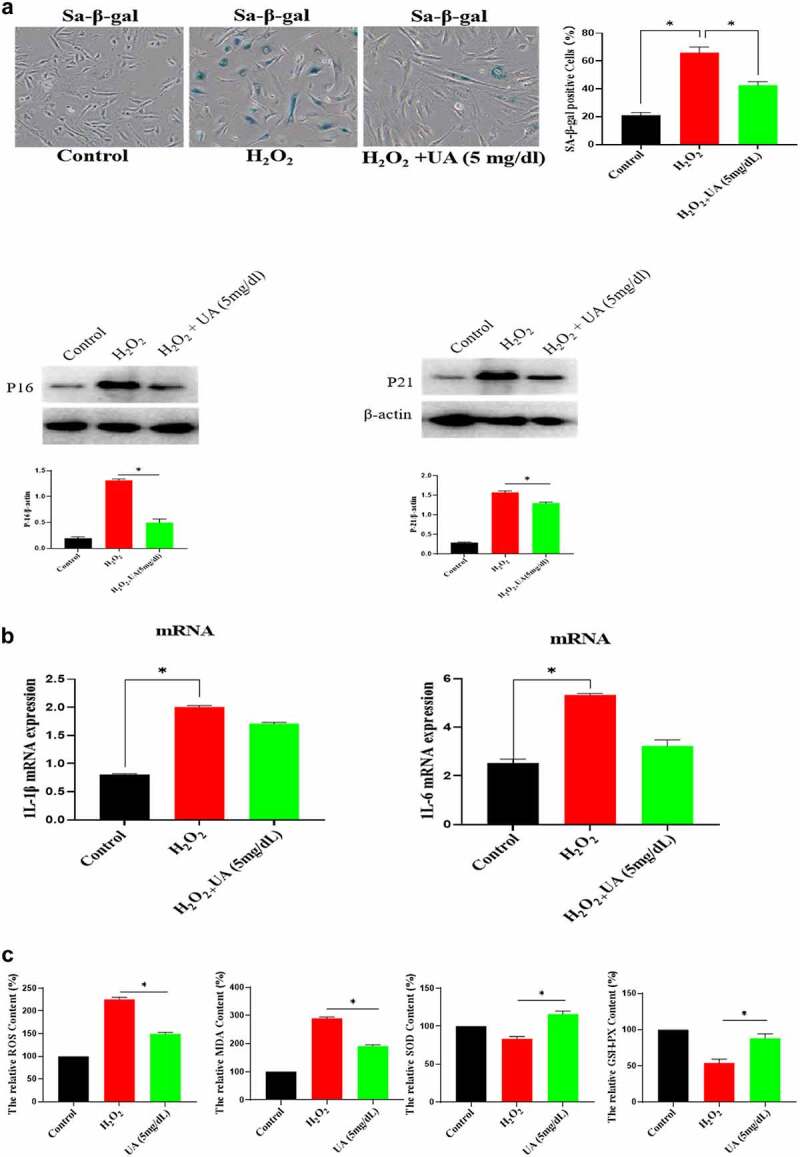
Effect of UA on biological activity of EGF/EGFR. a. Physiological concentration of UA (5 mg/dl) relieved the senescent HUVEC induced by H_2_O_2_ treatment. b-c. UA (5 mg/dl) down-regulated H_2_O_2_-induced oxidative stress and inflammation. d. physiological concentration of UA (5 mg/dl)restored the signaling ability of EGF/EGFR in senescent HUVEC. Data are presented as mean ± SD. Asterisks indicate statistically significant differences.

**Figure 6b.**
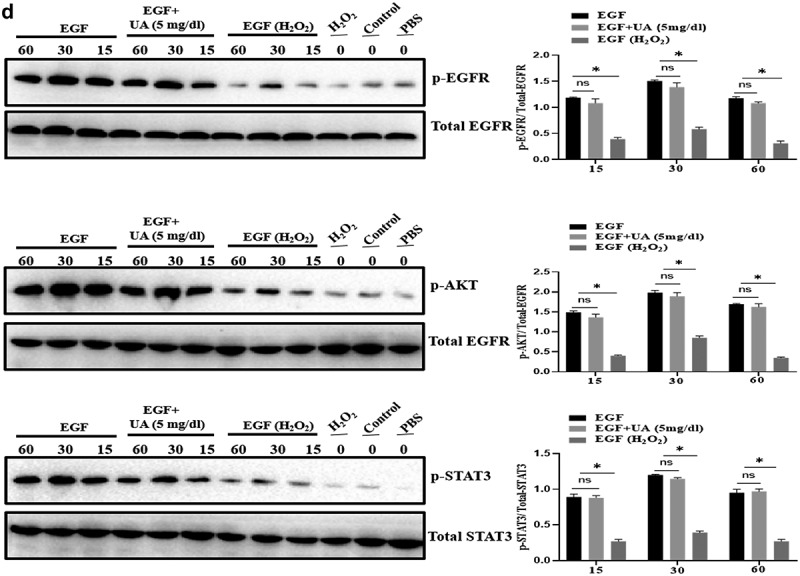
Continue

## Discussion

With the social and economic development and the improvement of living standards in China, people’s eating habits have changed, and hyperuricemia has occurred [14]. The incidence of hyperuricemia rate is increasing year by year [15]. More and more studies have found that high UA is a risk factor for various diseases (such as cardiovascular disease) [15]. Nowadays, hyperuricemia has become a chronic non-communicable epidemic disease. Similar to obesity, the incidence of hyperuricemia has been on the rise worldwide in recent decades. Elevated serum uric acid levels are associated with various cardiovascular diseases [16]. In addition, high level of UA can reduce NO synthesis in endothelial cells, causing endothelial dysfunction and increasing arterial stiffness. Hyperuricemia causes a lot of body damage, it has been reported that hyperuricemia is associated with the elevated risks of goat, diabetes, and chronic kidney [17]. Because UA exists in the blood circulatory system, UA has a significant effect on the function of vascular endothelium. Under normal physiological conditions, NO can participate in the regulation of vasomotor function, and the reduction of its concentration and biological activity is an important feature of endothelial dysfunction. eNOS is the precursor substance of NO synthesis, and the reduction of eNOS expression in the body can directly affect NO physiological function. Previous studies have shown that UA can reduce NO production, destroy its synthesis, and cause endothelial dysfunction [18].

EGF has many important regulatory effects on vascular endothelial cells, and a series of studies have shown that it is one of the most important molecules for regulating HUVEC proliferation [8,19]. But up to now, the cellular behavior of EGF/EGFR on HUVEC cells has not been revealed. In the current study, we have carried out a systematic study on this problem and found that EGF/EGFR could internalize into the cell and transport into the cell nuclei of HUVEC. Our further study found that the nuclear-localized EGFR is involved in the cell proliferation of HUVEC, which is a new discovery. The nuclear localization of EGFR has been widely reported in many types of cells, especially on tumor cells. Hung’s team has done a series of work in this field [20], who found that nuclear EGFR mainly has many important biological functions, such as regulating gene transcription and promoting cell proliferation. In addition, the nuclear localization of EGFR is closely related to tumor resistance [21].

An important scientific question is how does membrane-localized EGFR internalize from the cell membrane into the cytoplasm and transport into to the nucleus? Many studies have been done to explore this scientific issue. Actually, there are two main scientific questions that needs to be resolved: 1) how EGF/EGFR is endocytosed from the cell membrane into the cytoplasm. There are many studies to explore this issue. There are several endocytic routes, such as clathrin-mediated endocytosis and caveolin-mediated endocytosis; 2) The second scientific question is how does the internalized EGFR transports across the nuclear membrane. Study has shown that NUP358 which is localized at the nuclear pore complex (NPC) is involved EGFR’s nuclear translocation [20], and importin β is responsible for transporting EGFR to the nuclear membrane. In the current study, we found that the endocytosis of EGFR into cells is through clathrin- and caveolin-mediated pathway. Knockdown of NUP358 inhibited the nuclear transport of EGFR. These findings indicate that the EGFR enters into the cell nuclei via NUP358 mediation.

Next, we firstly explored the biological activity of the nuclear-localized EGF/EGFR system in HUVECs, and found that the nuclear localization of EGFR has important biological functions in HUVECs. Nuclear-localized EGFR is closely related to cell proliferation of HUVEC, and nuclear-localized EGFR is closely related to CyclinD1 expression. Similar findings have been reported in tumor-related cell models [22].

The current work shows that UA is a ‘double-edged sword’ which regulates EGF’s activity in the HUVECs, it is well known that HUVEC is an important cell model [23–25]. Our study found that high UA affected the biological function of EGF/EGFR system, and that high UA down-regulated EGFR-mediated signaling, and high UA inhibited EGFR’s nuclear localization. These experimental results indicated that high UA affects the biological activity of EGF/EGFR, but physiological concentration of UA has no effect on the activity of EGF/EGFR. We further studied the mechanism by which high UA down-regulated the EGFR signaling from the perspective of cell aging, and the results suggest that high UA could lead to HUVECs senescence (Aging is a biological process [26,27]), because high UA could promote oxidative stress and inflammation. More importantly, we found that physiological concentration of UA showed anti-oxidative stress effect. Recent studies have shown that physiological concentrations of UA can act as an antioxidant. In contrast, high concentration of UA can be converted into pro-oxidants, which will cause cell aging.

## Conclusions

In summary, our current work indicates that UA is a double-edged sword: high concentration of UA inhibits the biological activities of EGF/EGFR system on HUVEC. On the contrary, physiological concentration of UA has an anti-aging effect, which can increase the EGF/EGFR activities in the aged HUVECs.

## Limitation/short-coming of the study

The potential molecular mechanism of cell aging induced by high concentration uric acid needs to be further revealed.

## Future direction

Further exploration of the molecular mechanism by which anti-aging of uric acid in vivo is required in the future study.

## Supplementary Material

Supplemental Material

## Data Availability

All data canbe obtained by corresponding author upon the reasonable request.
