# Supplementary figures and images for: Uric acid, as a double-edged sword, affects the activity of epidermal growth factor (EGF) on human umbilical vein endothelial cells by regulating aging process
Source: Bioengineered. 2022 Feb 13;13(2):3877–95. doi: 10.1080/21655979.2022.2027172 (PMC8974203; doi:10.1080/21655979.2022.2027172)

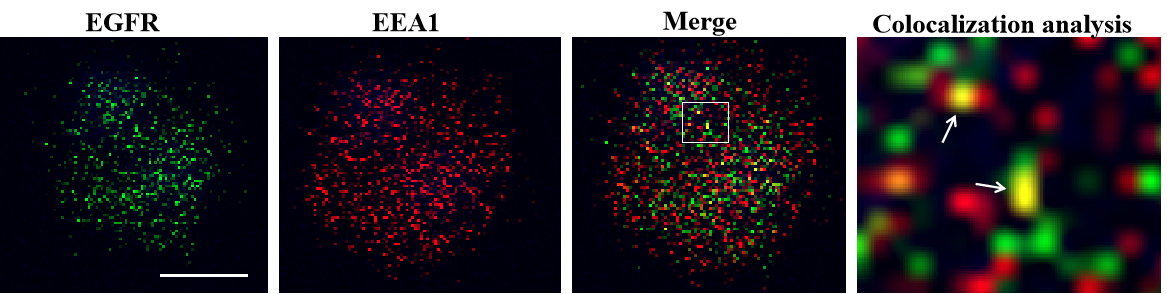

Supplement: Supplemental Material [file KBIE_A_2027172_SM8707.zip › supplementary/Supplementary_figure_1.docx]

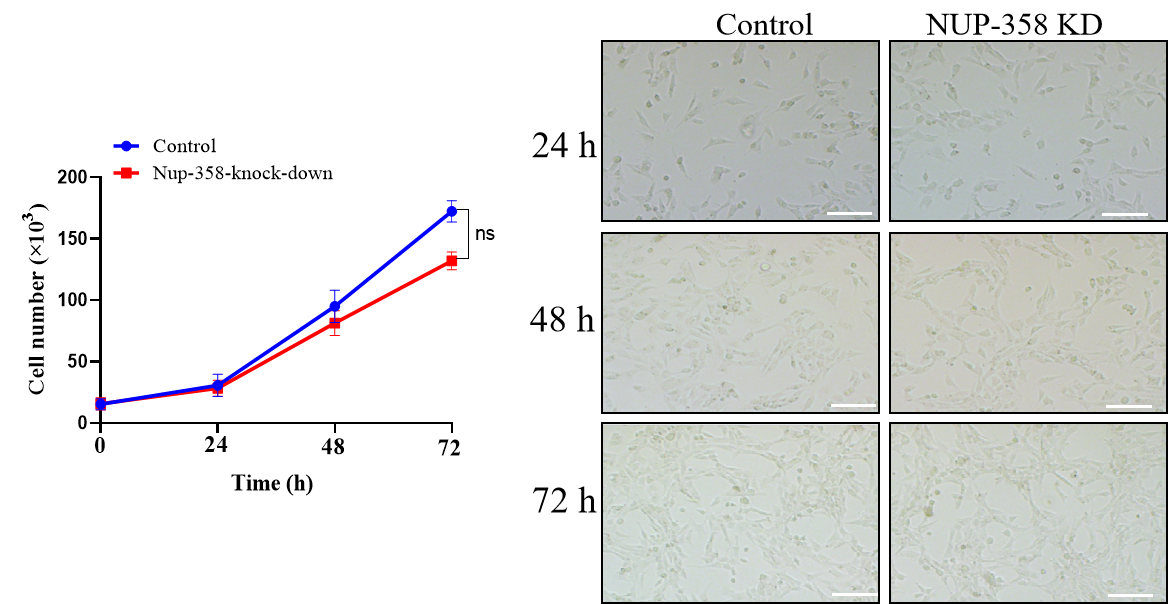

Supplement: Supplemental Material [file KBIE_A_2027172_SM8707.zip › supplementary/Supplementary_figure_2.docx]

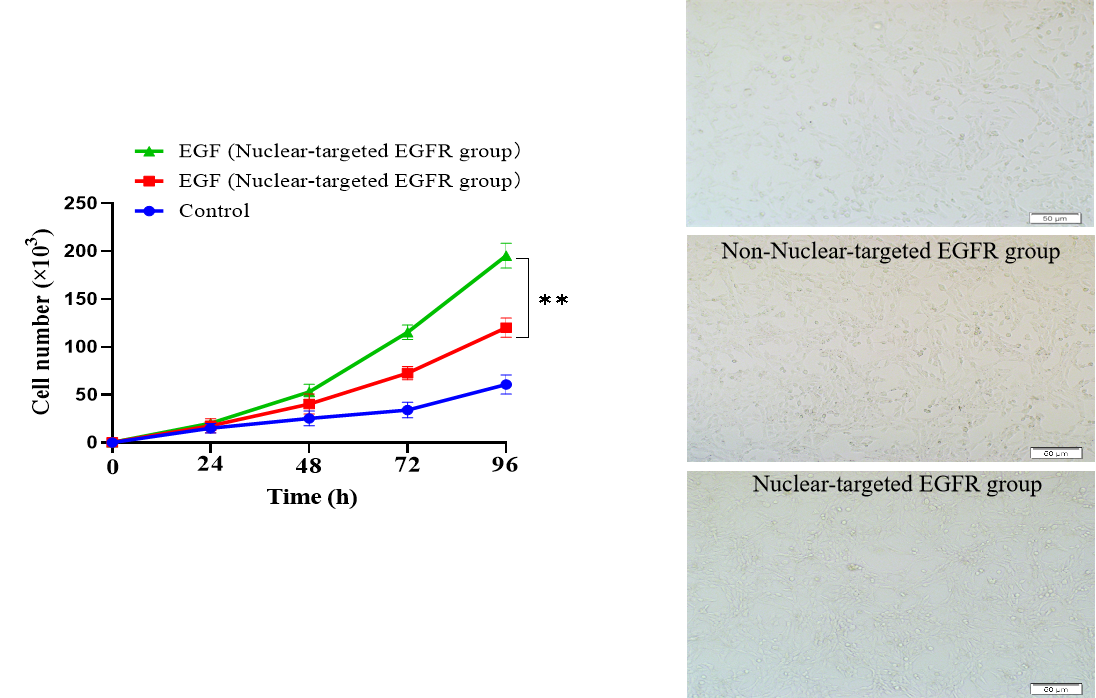

Supplement: Supplemental Material [file KBIE_A_2027172_SM8707.zip › supplementary/Supplementary_figure_3.docx]

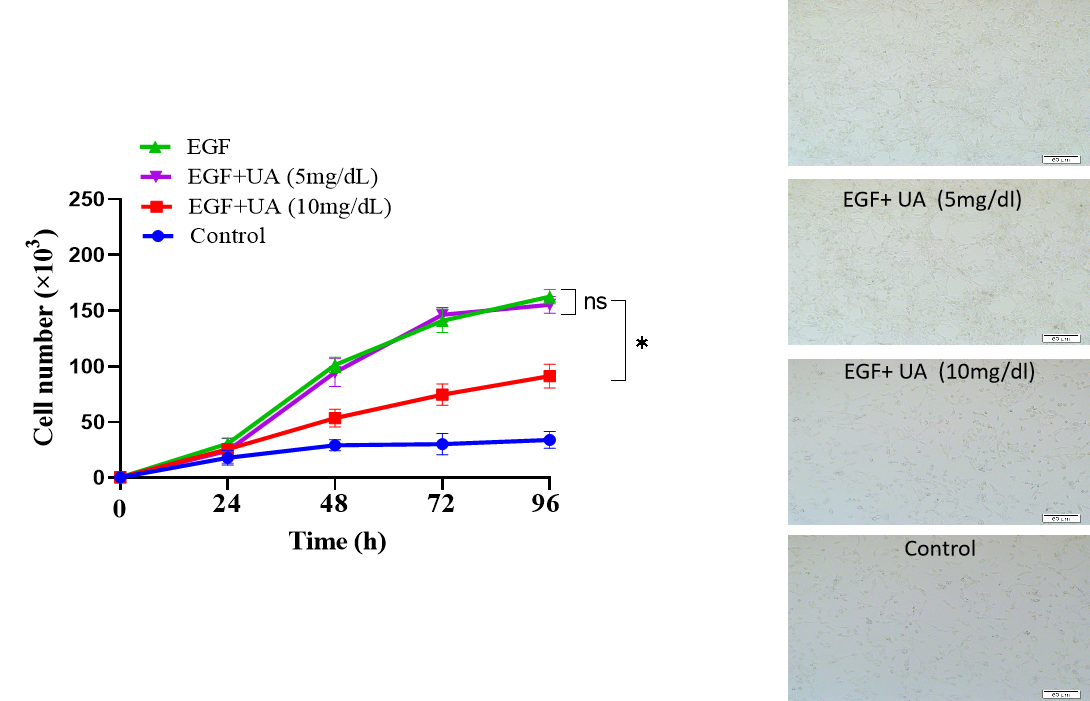

Supplement: Supplemental Material [file KBIE_A_2027172_SM8707.zip › supplementary/Supplementary_figure_4.docx]
